# Radio Frequency Compatibility Evaluation of S Band Navigation Signals for Future BeiDou

**DOI:** 10.3390/s17051039

**Published:** 2017-05-05

**Authors:** Yanbo Sun, Rui Xue, Danfeng Zhao, Dun Wang

**Affiliations:** 1 College of Information & Communication Engineering, Harbin Engineering University, Harbin 150001, China; sunyanbo12345@126.com (Y.S.); zhaodanfeng@hrbeu.edu.cn (D.Z.); 2 State Key Laboratory of Space-Ground Integrated Information Technology, Beijing 100086, China

**Keywords:** S band, satellite navigation, BeiDou, radio frequency compatibility

## Abstract

With L band frequency allocations for satellite navigation getting more crowded, S band (2483.5–2500 MHz) is already allocated for navigation services, where Globalstar broadcasts downlink communications to user terminals. The Indian Regional Navigation Satellite System (IRNSS) is transmitting navigation signals and Galileo exploits some potential signals in S band. Also, several candidate S band signals based on binary offset carrier (BOC), binary phase shift keying (BPSK), continuous phase modulation (CPM) and minimum shift keying-BOC (MSK-BOC) are suggested for BeiDou system (BDS). In quite narrow S band, mutual interference among these systems is inevitable, thus the compatibility issue is particularly significant for S band signal design. To explore desired S band signals for BDS, the paper firstly describes a comprehensive compatibility evaluation methods based on effective carrier-to-noise ratio degradation for acquisition and code tracking. Then a real simulation is established using space constellations, modulation schemes and received power. Finally, the worst mutual interference of BDS candidate signals with Galileo, IRNSS and Globalstar is calculated and compared. The results indicate that CPM signal is easier to allow peaceful coexistence of other systems with minimal mutual interference in S band compared to other BDS candidates.

## 1. Introduction

Along with the construction and development of global and regional navigation satellite systems, as well as space-based augmentation systems, it is predicted that more than 150 satellites and 400 signals will be present in space by 2030 [1,2]. In L band, such large amounts of signals will further deteriorate signal congestion situation and negatively impact the performance of signals sharing the same frequency band. A radical way for the evolution of navigation systems is to provide navigation services in new frequency bands. S band, between 2483.5 and 2500 MHz, has been allocated by the International Telecommunication Union (ITU) to navigation service [3]. The Globalstar communication satellite system also uses the same frequency slot to broadcast voice and data services with square root raised cosine (SRRC) for user terminals [4,5]. 

Because of smaller available bandwidth, S band signal performance hardly surpasses that of L band. However, the couple of S band signals with L band signal or Globalstar could improve positioning accuracy and timing performance, and promote the comprehensive performance of radio navigation services [6,7,8]. These excellent properties attract extensive attentions of system providers and researchers to S band signal structure for navigation systems, and modulation is one of key technologies that must be resolved during system design and updating. Nowadays, the Indian Regional Navigation Satellite System (IRNSS) is simultaneously broadcasting sine-phase binary offset carrier (BOCs(5,2)) and binary phase shift keying (BPSK(1)) in S band [9,10]. Also, Galileo exploits some possible signals in S band [5,6,11], including composite binary offset carrier (CBOC(6,1,1/11)), BPSK(1), BPSK(4) and BPSK(8). For BeiDou system (BDS), a few candidate signals based on BOCs(4,4), BPSK(8), continuous phase modulation (CPM(8)) and minimum shift keying-BOC (MSK-BOC(4,4)) are suggested by some researchers to provide an authorized service in S band [12,13,14]. However, in the quite finite S band of 16.5 MHz, mutual interference among different systems is inevitable. Thus, compatibility is a matter of great concern for system providers and has higher priority than other performance before the final confirmation of the new S band signals. To allow peaceful coexistence with minimal mutual interference among systems, radio frequency compatibility evaluation is particularly essential to design S band signals for future BDS. 

A quantity called the effective carrier-to-noise ratio degradation $\Delta {\left(C/N_{0}\right)}_{\mathrm{eff}}$ formulated by ITU-R can reflect the effects of interference among navigation systems at the receiver well [15]. The spectral separation coefficient (SSC) is an essential part of the $\Delta {\left(C/N_{0}\right)}_{\mathrm{eff}}$ and is accepted widely by the global navigation satellite system (GNSS) communities as an effective index to characterize compatibility. Nevertheless, the SSC is just appropriate to describe interference effects on signal acquisition rather than code tracking. To fill the gap, a code tracking spectral separation coefficient (CTSSC) is introduced for the evaluation of the interference level on code tracking loop [16]. To obtain more accurate compatibility assessments for the exploration of desired BDS signals allowing peaceful coexistence with other systems in S band, comprehensive compatibility evaluation methods based on the $\Delta {\left(C/N_{0}\right)}_{\mathrm{eff}}$ using SSC and CTSSC are firstly described and derived from the perspective of output SNIR and code tracking error variance for both scenarios of acquisition and code tracking respectively. A real simulation is established using completed space constellation models of navigation and Globalstar systems, modulation schemes and received power, though it is tough work to calculate each interfering case for different static users on the earth’s surface at every moment. The worst results of compatibility between BDS candidate S band signals and other systems are calculated in simulation using the 10°×10° grid for longitude and latitude and sampling time step of 1 minute during a time period of 10 days. 

The rest of this paper is organized as follows. Section 2 presents complete compatibility evaluation methods based on the $\Delta {\left(C/N_{0}\right)}_{\mathrm{eff}}$ for both scenarios of the acquisition and code tracking. Signal parameters and space constellation models of BDS, IRNSS, Galileo and Globalstar are established in Section 3. The real simulation considering the worst situation on compatibility of BDS candidate signals with other system signals in S band is carried out in Section 4. Finally, we conclude the paper in Section 5.

## 2. Radio Frequency Compatibility Evaluation Methods 

### 2.1. Compatibility Evaluation Method for Acquisition 

Acquisition refers to the coarse estimation of code phase and Doppler by the prompt correlation of received signal with hypotheses reference signal. The output signal-to-noise-plus-interference ratio (SNIR) of prompt correlator can describe signal acquisition performance well. For noncoherent processing, the output SNIR at the prompt correlator is expressed as [17,18]

(1) $$\rho_{n}=\frac{T\frac{C}{N_{0}}{\left(\int_{-B/2}^{B/2}G_{s}\left(f\right)df\right)}^{2}}{\int_{-B/2}^{B/2}G_{s}\left(f\right)df+\frac{C_{J}}{N_{0}}\int_{-B/2}^{B/2}G_{s}\left(f\right)G_{J}\left(f\right)df},$$

where the $G_{s}(f)$ and $G_{J}(f)$ are respectively the power spectrum density (PSD) of desired signal and interfering signal, normalized to unit power over infinite transmission bandwidth, the C and $C_{J}$ are receiver antenna output power of desired signal and interfering signal, B is the receiver pre-filtering bandwidth, T is the integration time and $N_{0}$ is the PSD of white noise taking value of −204 dBW/Hz. The SSC is the inner product of PSDs between desired and interfering signals, defined as

(2) $$\chi_{J}^{s}=\int_{-B/2}^{B/2}G_{s}(f)G_{J}(f)df.$$

As reported in Reference [19], when the white noise exists alone, the received $C/N_{0}$ is simply equivalent to the effective carrier-to-noise ratio, resulting in

(3) $${\left(\frac{C}{N_{0}}\right)}_{\mathrm{eff},\mathrm{SSC}}=\frac{\rho_{n}}{T\int_{-B/2}^{B/2}G_{s}\left(f\right)df}.$$

When the both of white noise and non-white interference are present, the general expression of the ${\left(C/N_{0}\right)}_{\mathrm{eff},\mathrm{SSC}}$ can be derived by substituting Equation (1) into Equation (3), i.e.,

(4) $${\left(\frac{C}{N_{0}}\right)}_{\mathrm{eff},\mathrm{SSC}}=\frac{C}{N_{0}+\frac{C_{J}\int_{-B/2}^{B/2}G_{s}\left(f\right)G_{J}\left(f\right)df}{\int_{-B/2}^{B/2}G_{s}\left(f\right)df}}.$$

Obviously, the ${\left(C/N_{0}\right)}_{\mathrm{eff},\mathrm{SSC}}$ can be treated as the other interpretation of output SNIR to clarify how the combination of noise and interference affects signal acquisition. In real environment, several space constellations may coexist in same frequency band, then the intersystem and intrasystem interference must be considered for more accurate compatibility evaluation. Thus, the ${\left(C/N_{0}\right)}_{\mathrm{eff},\mathrm{SSC}}$ can further be deduced as

(5) $$\begin{array}{ll} {\left(\frac{C}{N_{0}}\right)}_{\mathrm{eff},\mathrm{SSC}} & =\frac{C}{N_{0}+\frac{\sum_{j}^{N_{\mathrm{satel}}^{\mathrm{intra}}\left(t\right)}\sum_{k}^{N_{\mathrm{signal},j}^{\mathrm{intra}}}C_{j,k}^{\mathrm{intra}}\int_{-B/2}^{B/2}G_{s}\left(f\right)G_{j,k}^{\mathrm{intra}}\left(f\right)df}{\int_{-B/2}^{B/2}G_{s}\left(f\right)df}+\frac{\sum_{i}^{N_{\mathrm{inter}}}\sum_{j}^{N_{\mathrm{satel},i}^{\mathrm{inter}}\left(t\right)}\sum_{k}^{N_{\mathrm{signal},i,j}^{\mathrm{inter}}}C_{i,j,k}^{\mathrm{inter}}\int_{-B/2}^{B/2}G_{s}\left(f\right)G_{i,j,k}^{\mathrm{inter}}\left(f\right)df}{\int_{-B/2}^{B/2}G_{s}\left(f\right)df}}\\ & =\frac{C}{N_{0}+I_{\mathrm{intra}}^{\mathrm{SSC}}\left(t\right)+I_{\mathrm{inter}}^{\mathrm{SSC}}\left(t\right)} \end{array},$$

where $N_{\mathrm{satel}}^{\mathrm{intra}}\left(t\right)$ is the number of intrasystem visible satellites from same constellation as desired signal at t moment, $N_{\mathrm{signal},j}^{\mathrm{intra}}$ is the number of interfering signals transmitted by the *j*th intrasystem satellite, $C_{j,k}^{\mathrm{intra}}$ and $G_{j,k}^{\mathrm{intra}}\left(f\right)$ are respectively the receiver antenna output power and PSD of the *k*th interfering signal of the *j*th intrasystem satellite, $N_{\mathrm{inter}}$ is the number of intersystems using other space constellations, $N_{\mathrm{satel},i}^{\mathrm{inter}}\left(t\right)$ is the visible satellite number of the *i*th intersystem at t moment, $N_{\mathrm{signal},\iota ,j}^{\mathrm{inter}}$ is the signal number transmitted by the *j*th satellite belonging to the *i*th intersystem, and $C_{i,j,k}^{\mathrm{inter}}$ and $G_{i,j,k}^{\mathrm{inter}}\left(f\right)$ are the receiver antenna output power and PSD of the *k*th interfering signal from the *j*th satellite of the *i*th intersystem. Here, the $I_{\mathrm{intra}}^{\mathrm{SSC}}\left(t\right)$ and $I_{\mathrm{inter}}^{\mathrm{SSC}}\left(t\right)$ are separately equivalent PSD for the intrasystem and intersystem interference based on the SSC at t moment, and the corresponding aggregate equivalent PSD of interference $I_{\mathrm{total}}^{\mathrm{SSC}}\left(t\right)$ is the sum of the $I_{\mathrm{intra}}^{\mathrm{SSC}}\left(t\right)$ and $I_{\mathrm{inter}}^{\mathrm{SSC}}\left(t\right)$. To better understand these concepts, for example, we assume only IRNSS, Globalstar and Galileo are present in S band, their signal parameters are described in introduction section. If the Galileo broadcasts BPSK(1) in S band as the desired signal, then the aggregate equivalent PSD of interference $I_{\mathrm{total}}$ can be expressed by

(6) $$I_{\mathrm{total}}=I_{\mathrm{intra}}+I_{\mathrm{inter}},$$

with

(7) $$\{\begin{array}{l} I_{\mathrm{intra}}=I_{\mathrm{BPSK}\left(1\right),\mathrm{others}}^{\mathrm{Galileo}}\\ I_{\mathrm{inter}}=I_{\mathrm{SRRC}}^{\mathrm{Globalstar}}+I_{\mathrm{BPSK}\left(1\right)}^{\mathrm{IRNSS}}+I_{\mathrm{BOCs}\left(5,2\right)}^{\mathrm{IRNSS}} \end{array},$$

where the $I_{\mathrm{BPSK}\left(1\right),\mathrm{others}}^{\mathrm{Galileo}}$ represents the equivalent PSD of BPSK(1) interfering signals from other Galileo visible satellites, not including that one transmitting desired BPSK(1) signal. Likewise, the $I_{\mathrm{BOCs}\left(5,2\right)}^{\mathrm{IRNSS}}$ denotes the equivalent PSD of BOCs(5,2) interfering signals from all visible satellites of IRNSS system. 

To reflect the relative weakening of a desired signal due to the intrasystem or intersystem interference, an indicator called effective carrier-to-noise ratio degradation $\Delta {\left(C/N_{0}\right)}_{\mathrm{eff}}$ is introduced by ITU-R [15]. The $\Delta {\left(C/N_{0}\right)}_{\mathrm{eff}}$ of desired signal induced by the intrasystem interference in acquisition is derived by

(8) $$\Delta {\left(C/N_{0}\right)}_{\mathrm{eff},\mathrm{SSC}}^{\mathrm{intra}}=\frac{C}{N_{0}}/\frac{C}{N_{0}+I_{\mathrm{intra}}^{\mathrm{SSC}}\left(t\right)}=1+\frac{I_{\mathrm{intra}}^{\mathrm{SSC}}\left(t\right)}{N_{0}}.$$

Similarly, the $\Delta {\left(C/N_{0}\right)}_{\mathrm{eff}}$ of desired signal induced by the intersystem interference in acquisition is given by

(9) $$\Delta {\left(C/N_{0}\right)}_{\mathrm{eff},\mathrm{SSC}}^{\mathrm{inter}}=\frac{C}{N_{0}+I_{\mathrm{intra}}^{\mathrm{SSC}}\left(t\right)}/\frac{C}{N_{0}+I_{\mathrm{intra}}^{\mathrm{SSC}}\left(t\right)+I_{\mathrm{inter}}^{\mathrm{SSC}}\left(t\right)}=1+\frac{I_{\mathrm{inter}}^{\mathrm{SSC}}\left(t\right)}{N_{0}+I_{\mathrm{intra}}^{\mathrm{SSC}}\left(t\right)}.$$

### 2.2. Compatibility Evaluation Method for Code Tracking 

Code tracking is used for the accurate estimation of code phase and requires two correlator channels namely early and late ones. A general method for measuring code tracking performance is to estimate the variance of smoothed time of arrival (TOA). When the noncoherent discriminator is employed using the delay between early and late of d, the variance of smoothed TOA is expressed as [17]

(10) $$\begin{array}{ll} \sigma_{non}^{2} & =\left(\frac{B_{L}\left(1-0.5B_{L}T\right)\int_{-B/2}^{B/2}G_{s}(f)\sin^{2}\left(\pi fd\right)df}{{\left(2\pi \right)}^{2}\frac{C}{N_{0}}{\left(\int_{-B/2}^{B/2}fG_{s}\left(f\right)\sin\left(\pi fd\right)df\right)}^{2}}+\frac{B_{L}\left(1-0.5B_{L}T\right)C_{J}\int_{-B/2}^{B/2}G_{s}(f)G_{J}\left(f\right)\sin^{2}\left(\pi fd\right)df}{{\left(2\pi \right)}^{2}C{\left(\int_{-B/2}^{B/2}fG_{s}\left(f\right)\sin\left(\pi fd\right)df\right)}^{2}}\right)\\ & \times \left(1+\frac{\int_{-B/2}^{B/2}G_{s}(f)\cos^{2}\left(\pi fd\right)df}{T\frac{C}{N_{0}}{\left(\int_{-B/2}^{B/2}fG_{s}\left(f\right)\cos\left(\pi fd\right)df\right)}^{2}}+\frac{C_{J}\int_{-B/2}^{B/2}G_{s}(f)G_{J}\left(f\right)\cos^{2}\left(\pi fd\right)df}{TC{\left(\int_{-B/2}^{B/2}fG_{s}\left(f\right)\cos\left(\pi fd\right)df\right)}^{2}}\right)\\ & =\sigma_{coh}^{2}\times \vartheta \end{array},$$

where $B_{L}$ is the loop bandwidth. Through Equation (10), we can observe that the carrier-to-noise ratio has very significant impacts on the error variance, so it can also be indirectly regarded as a measure of code tracking performance. Besides, the noncoherent error variance is the product of coherent error variance $\sigma_{coh}^{2}$ [20] and squaring loss ϑ greater than 1. The ϑ is very close to 1 for the usual range of $C/N_{0}$ greater than 30 dB-Hz [16], then the both of the $\sigma_{non}^{2}$ and $\sigma_{coh}^{2}$ are approximately equivalent. To attain the ${\left(C/N_{0}\right)}_{\mathrm{eff}}$ for code tracking, the error variance in white noise is obtained when the $C_{J}$ equals 0, i.e.,

(11) $$\sigma_{coh}^{2}=\frac{B_{L}\left(1-0.5B_{L}T\right)\int_{-B/2}^{B/2}G_{s}(f)\sin^{2}\left(\pi fd\right)df}{{\left(2\pi \right)}^{2}\frac{C}{N_{0}}{\left(\int_{-B/2}^{B/2}fG\left(f\right)\sin\left(\pi fd\right)df\right)}^{2}}.$$

Since the received $C/N_{0}$ simply equals the ${\left(C/N_{0}\right)}_{\mathrm{eff}}$ in white noise, then the ${\left(C/N_{0}\right)}_{\mathrm{eff}}$ for code tracking is derived as

(12) $${\left(\frac{C}{N_{0}}\right)}_{\mathrm{eff},\mathrm{CTSSC}}=\frac{B_{L}\left(1-0.5B_{L}T\right)\int_{-B/2}^{B/2}G_{s}(f)\sin^{2}\left(\pi fd\right)df}{{\left(2\pi \right)}^{2}\sigma_{coh}^{2}{\left(\int_{-B/2}^{B/2}fG\left(f\right)\sin\left(\pi fd\right)df\right)}^{2}},$$

When the white noise and non-white interference signal exist, we substitute Equation (10) into Equation (12) and attain the corresponding ${\left(C/N_{0}\right)}_{\mathrm{eff},\mathrm{CTSSC}}$ as

(13) $${\left(\frac{C}{N_{0}}\right)}_{\mathrm{eff},\mathrm{CTSSC}}=\frac{C}{N_{0}+\frac{C_{J}\int_{-B/2}^{B/2}G_{s}(f)G_{J}\left(f\right)\sin^{2}\left(\pi fd\right)df}{\int_{-B/2}^{B/2}G_{s}\left(f\right)\sin^{2}\left(\pi fd\right)df}}$$

with the CTSSC defined as

(14) $$\kappa_{J}^{s}=\frac{\int_{-B/2}^{B/2}G_{s}(f)G_{J}\left(f\right)\sin^{2}\left(\pi fd\right)df}{\int_{-B/2}^{B/2}G_{s}\left(f\right)\sin^{2}\left(\pi fd\right)df}.$$

When considering multiple space constellations, the ${\left(C/N_{0}\right)}_{\mathrm{eff},\mathrm{CTSSC}}$ with the intersystem and intrasystem interference can be deduced as

(15) $$\begin{array}{ll} {\left(\frac{C}{N_{0}}\right)}_{\mathrm{eff},\mathrm{CTSSC}} & =\frac{C}{N_{0}+\sum_{j}^{N_{\mathrm{satel}}^{\mathrm{intra}}\left(t\right)}\sum_{k}^{N_{\mathrm{signal},j}^{\mathrm{intra}}}C_{j,k}^{\mathrm{intra}}\kappa_{\mathrm{intra}\left(j,k\right)}^{s}+\sum_{i}^{N_{\mathrm{inter}}}\sum_{j}^{N_{\mathrm{satel},i}^{\mathrm{inter}}\left(t\right)}\sum_{k}^{N_{\mathrm{signal},i,j}^{\mathrm{inter}}}C_{i,j,k}^{\mathrm{inter}}\kappa_{\mathrm{inter}\left(i,j,k\right)}^{s}}\\ & =\frac{C}{N_{0}+I_{\mathrm{intra}}^{\mathrm{CTSSC}}\left(t,d\right)+I_{\mathrm{inter}}^{\mathrm{CTSSC}}\left(t,d\right)} \end{array},$$

where $\kappa_{j,k}^{s}$ are the CTSSC of desired signal with the *k*th interfering signal transmitted by the *j*th intrasystem satellite, $\kappa_{i,j,k}^{s}$ is the CTSSC of desired signal with the *k*th interfering signal from the *j*th satellite of the *i*th intersystem. Similarly, the $I_{\mathrm{intra}}^{\mathrm{CTSSC}}\left(t,d\right)$ and $I_{\mathrm{inter}}^{\mathrm{CTSSC}}\left(t,d\right)$ are separately equivalent PSD for the intrasystem and intersystem interference based on the CTSSC using a certain d at t moment. To investigate the impacts of intrasystem or intersystem interference on code tracking performance of a desired signal, similar evaluation methods with those of signal acquisition based on the $\Delta {\left(C/N_{0}\right)}_{\mathrm{eff}}$ are presented as follows

(16) $$\Delta {\left(C/N_{0}\right)}_{\mathrm{eff},\mathrm{CTSSC}}^{\mathrm{intra}}=\frac{C}{N_{0}}/\frac{C}{N_{0}+I_{\mathrm{intra}}^{\mathrm{CTSSC}}\left(t,d\right)}=1+\frac{I_{\mathrm{intra}}^{\mathrm{CTSSC}}\left(t,d\right)}{N_{0}},$$

(17) $$\Delta {\left(C/N_{0}\right)}_{\mathrm{eff},\mathrm{CTSSC}}^{\mathrm{inter}}=\frac{C}{N_{0}+I_{\mathrm{intra}}^{\mathrm{CTSSC}}\left(t,d\right)}/\frac{C}{N_{0}+I_{\mathrm{intra}}^{\mathrm{CTSSC}}\left(t,d\right)+I_{\mathrm{inter}}^{\mathrm{CTSSC}}\left(t,d\right)}=1+\frac{I_{\mathrm{inter}}^{\mathrm{CTSSC}}\left(t,d\right)}{N_{0}+I_{\mathrm{intra}}^{\mathrm{CTSSC}}\left(t,d\right)}.$$

## 3. Space Constellation and Signal Parameters

### 3.1. Space Constellation

To attain more accurate compatibility evaluation of S band signals, the complete space constellation models of Galileo, BDS, IRNSS and Globalstar are established in this paper. Their detailed space constellation parameters are summarized in Table 1. 

The IRNSS space segment will eventually comprise seven satellites. Three satellites in geostationary orbits (GSOs) are placed at 32.5° E, 83° E, and 131.5° E and four satellites are in inclined geosynchronous orbits (IGSOs) where the first two satellites operate at 55° E with an inclination of 29° with respect to the equator and the other two satellites have their longitude crossing at 111.75° E [21]. The Globalstar is deploying a second-generation constellation containing thirty-two low earth-orbiting (LEO) satellites. These satellites are in prograde circular orbits at 52° inclination on eight orbit planes spaced equally [22,23]. According to the interface control documents (ICD) of Galileo [24], the fully deployed Galileo system will consist of 27 operational satellites, positioned in three circular medium earth orbit (MEO) planes at an inclination of the orbital planes of 56° with reference to the equatorial plane. The BDS will accomplish a fully deployed constellation of 35 satellites by 2020, including five GSO satellites, twenty-seven MEO satellites and three IGSO satellites [25,26,27]. The GSO satellites are positioned at 58.75° E, 80° E, 110.5° E, 140° E and 160° E respectively. The MEO satellites are operating with an inclination of 55° relative to the equatorial plane. The three IGSO satellites work at 118° E using an inclination of 55°. 

### 3.2. S Band Signals 

**Figure 1:**
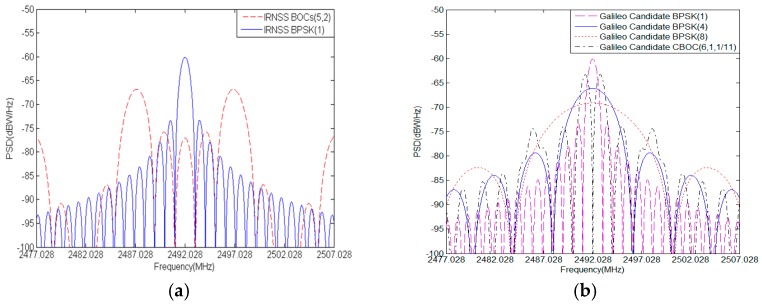

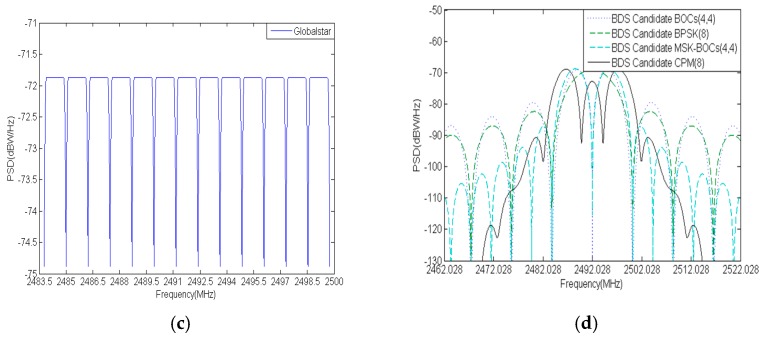
S band Signal PSD: (**a**) IRNSS; (**b**) Galileo candidates; (**c**) Globalstar; (**d**) BDS candidates.

In the S band, the IRNSS is simultaneously broadcasting BPSK(1) and BOCs(5,2) for open and authorized services respectively [9,10]. The Galileo also exploits a series of S band candidates such as CBOC(6,1,1/11), BPSK(1), BPSK(4), and BPSK(8) for introducing alternate and complementary capabilities to some services in operation or under deployment [5,6,11]. The Globalstar employs the same S band frequency slot to transmit downlink signals by multi-beam antennas allowing frequency reutilization [5]. Each beam contains 13 frequency division multiple access (FDMA) channels with each channel taking up 1.23 MHz wide. Code division multiple access (CDMA) with a chipping rate of 1.2288 Mcps is implemented inside every FDMA channel. Before modulation of the carrier, the Globalstar signal is filtered by SRRC filter with roll-off factor ρ of 0.2, then Globalstar signal PSD of the each beam can de expressed by

(18) $$G_{Globalstar}\left(f\right)=\sum_{k=-6}^{6}G_{SRRC}^{k}\left(f\right)$$

where PSD of SRRC at the *k*th FDMA channel is indicated as

(19) $$G_{SRRC}^{k}\left(f\right)=\left\{ \begin{array}{ll} 1, & \left|f-kB_{f}\right|\leq \frac{f_{c}}{2}\left(1-\rho \right)\\ 0, & \left|f-kB_{f}\right|\leq \frac{f_{c}}{2}\left(1+\rho \right)\\ g\left(f\right), & \frac{f_{c}}{2}\left(1-\rho \right)\leq \left|f-kB\right|\leq \frac{f_{c}}{2}\left(1+\rho \right) \end{array}\right.,$$

with

(20) $$g\left(f\right)=\frac{1+\cos\left(\frac{\pi }{\rho f_{c}}\left(\left|f\right|-\frac{\left(1-\rho \right)f_{c}}{2}-kB_{f}\right)\right)}{2}$$

where the $f_{c}$ is a chip rate of 1.2288 Mcps, $B_{f}$ is a FDMA bandwidth of 1.23 MHz and ρ is a roll-off factor of 0.2. The S band signal PSDs of IRNSS, Galileo candidate and Globalstar are shown in Figure 1a–c. 

To take full advantages of S band’s superiorities and reserve resources for BDS, signal modulation schemes in S band have attracted extensive research attentions. The legacy modulations of BPSK(8) and BOCs(4,4) are suggested for BDS in S band [12]. Besides, a promising modulation called CPM with a chip rate of 8.184 Mcps, frequency pulse g(t) of RC, modulation order M=2 and pulse length L=2 denoted by CPM(8) and MSK-BOCs(4,4) are also recommended as candidate S band BDS signals [13,14], because their inherent properties of constant envelope and phase continuity contribute to greatly reducing the nonlinear distortion due to the saturating characteristic of the high power amplifier and linear bandpass distortion due to nonideal bandpass characteristic in the satellite navigation applications. The PSD of CPM can be expressed as

(21) $$\begin{array}{ll} P(f) & =2\{\int_{0}^{LT}ℜ(\tau )\cos2\pi f\tau d\tau +\frac{1-\psi (jh)\cos2\pi fT}{1+\psi^{2}(jh)-2\psi (jh)\cos2\pi fT}\cdot \int_{LT}^{(L+1)T}ℜ(\tau )\cos2\pi f\tau d\tau \\ & -\frac{\psi (jh)\sin2\pi fT}{1+\psi^{2}(jh)-2\psi (jh)\cos2\pi fT}\cdot \int_{LT}^{(L+1)T}ℜ(\tau )\sin2\pi f\tau d\tau \} \end{array},$$

with

(22) ψ(jh)=sinMπh/Msinπh,

where the ℜ(τ) refers to the autocorrelation function of CPM, i.e.,

(23) $$ℜ(\tau )=\frac{1}{T}\int_{0}^{T}\prod_{k=1-L}^{\left\lfloor \tau /T\right\rfloor }\frac{1}{M}\frac{\sin2\pi hM[q(t+\tau -kT)-q(t-kT)]}{\sin2\pi h[q(t+\tau -kT)-q(t-kT)]}dt,$$

where ⌊⋅⌋ is the floor rounding operator. The PSD of MSK-BOC is written as

(24) $$G_{\mathrm{MSK}-\mathrm{BOCs}(n,m)}\left(f\right)=\left\{ \begin{array}{l} \frac{2f_{s}^{2}f_{c}\sin\left(\pi f/f_{c}\right)}{\pi^{2}{\left(f_{s}^{2}-f^{2}\right)}^{2}},2n/m\;\mathrm{is}\;\mathrm{even}\\ \frac{2f_{s}^{2}f_{c}\cos\left(\pi f/f_{c}\right)}{\pi^{2}{\left(f_{s}^{2}-f^{2}\right)}^{2}},2n/m\;\mathrm{is}\;\mathrm{odd} \end{array}\right.,$$

where the $f_{s}$ is subcarrier frequency of *n ×* 1.023 MHz and the $f_{c}$ is chip rate of *m ×* 1.023 MHz. The Figure 1d shows all S band candidates for BDS with similar spectrum occupation in main lobe. In views of the severe spectrum overlap in S band, the compatibility is a particularly critical issue that must be considered for BDS signal design in S band. 

## 4. Radio Frequency Compatibility Evaluation

**Figure 2:**
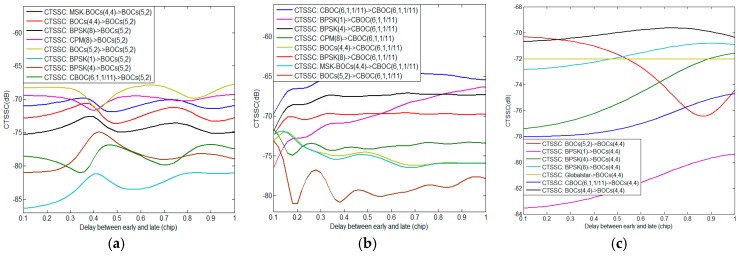

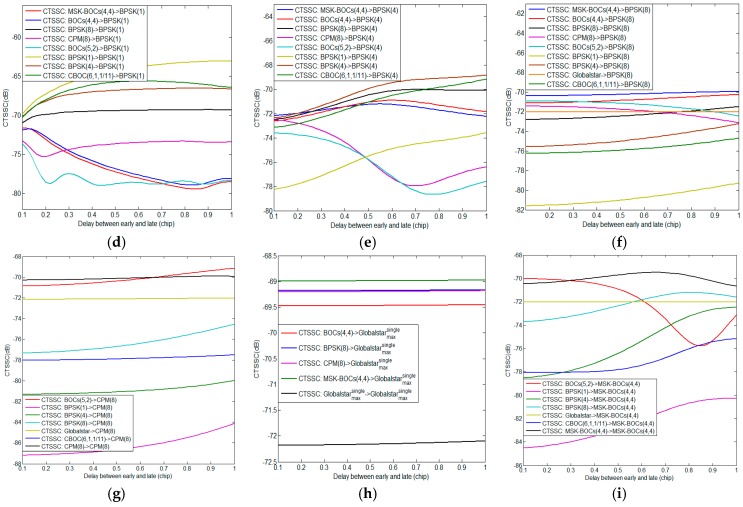
The CTSSC of (**a**) BOCs(5,2); (**b**) CBOC(6,1,1/11); (**c**) BOCs(4,4); (**d**) BPSK(1); (**e**) BPSK(4); (**f**) BPSK(8); (**g**) CPM(8); (**h**) ${\mathrm{Globalstar}}_{\max}^{\mathrm{single}}$ and (**i**) MSK-BOCs(4,4) with other interference signals.

This section provides compatibility evaluation results of BDS candidate signals with interfering signals from other systems in S band. Corresponding simulation parameters are shown in Table 2. Based on the previous analysis, the SSC and CTSSC are the essential quantities for the calculation of $\Delta {\left(C/N_{0}\right)}_{\mathrm{eff}}$ in both scenarios of acquisition and code tracking. The Table 3 and Figure 2 respectively report the SSC and CTSSC of each S band signal as desired signal with other interference signals, where ${\mathrm{Globalstar}}_{\max}^{\mathrm{single}}$ means the single FDMA Globalstar signal corresponding to the maximum SSC and CTSSC with interference signal, and the delay between early and late varies from 0.1 to 1 chip of desired signal. 

From Table 3 and Figure 2, it clearly appears that each CTSSC is different from corresponding SSC, which implies interfering signals have disparate impacts on same desired signal in terms of acquisition and code tracking. Besides the CTSSC is very sensitive to *d* due to the $\sin^{2}\left(\pi fd\right)$ function that would produce large amounts of compatibility evaluation results in code tracking using various *d* for each sampling time. Here we analyze the worst situation considering the minimal desired signal power, maximum interfering signal power as well as the maximum equivalent PSD of the intersystem or intrasystem interference. Then the worst $\Delta {\left(C/N_{0}\right)}_{\mathrm{eff}}$ of desired signal induced by the intrasystem interference in acquisition is expressed by

(25) $$\Delta {\left(C/N_{0}\right)}_{\mathrm{eff},\mathrm{SSC}}^{\mathrm{intra},\mathrm{worst}}=\underset{\arg\max\left(I_{\mathrm{intra},\mathrm{worst}}^{\mathrm{SSC}}\left(t\right)\right),C_{j,k}^{\mathrm{intra}}=C_{j,k}^{\mathrm{intra},\max}}{\Delta {\left(C/N_{0}\right)}_{\mathrm{eff},\mathrm{SSC}}^{\mathrm{intra}}},$$

where $C_{j,k}^{\mathrm{intra},\max}$ is the maximum receiver antenna output power of the *k*th interfering signal of the *j*th intrasystem satellite. The worst $\Delta {\left(C/N_{0}\right)}_{\mathrm{eff}}$ of desired signal induced by the intersystem interference in acquisition is expressed by

(26) $$\Delta {\left(C/N_{0}\right)}_{\mathrm{eff},\mathrm{SSC}}^{\mathrm{inter},\mathrm{worst}}=\underset{\arg\max\left(I_{\mathrm{inter},\mathrm{worst}}^{\mathrm{SSC}}\left(t\right)\right),C_{i,j,k}^{\mathrm{inter}}=C_{i,j,k}^{\mathrm{inter},\max}}{\Delta {\left(C/N_{0}\right)}_{\mathrm{eff},\mathrm{SSC}}^{\mathrm{inter}}},$$

where $C_{i,j,k}^{\mathrm{inter},\max}$ is the maximum receiver antenna output power of the *k*th interfering signal from the *j*th intrasystem satellite of the *i*th intersystem. Likewise, the worst $\Delta {\left(C/N_{0}\right)}_{\mathrm{eff}}$ of desired signal induced by the intrasystem and intersystem interference in code tracking are expressed respectively as follows

(27) $$\Delta {\left(C/N_{0}\right)}_{\mathrm{eff},\mathrm{CTSSC}}^{\mathrm{intra},\mathrm{worst}}=\underset{\arg\max\left(I_{\mathrm{intra},\mathrm{worst}}^{\mathrm{CTSSC}}\left(t,d\right)\right),C_{j,k}^{\mathrm{intra}}=C_{j,k}^{\mathrm{intra},\max}}{\Delta {\left(C/N_{0}\right)}_{\mathrm{eff},\mathrm{CTSSC}}^{\mathrm{intra}}},$$

(28) $$\Delta {\left(C/N_{0}\right)}_{\mathrm{eff},\mathrm{CTSSC}}^{\mathrm{inter},\mathrm{worst}}=\underset{\arg\max\left(I_{\mathrm{inter},\mathrm{worst}}^{\mathrm{CTSSC}}\left(t,d\right)\right),C_{i,j,k}^{\mathrm{inter}}=C_{i,j,k}^{\mathrm{inter},\max}}{\Delta {\left(C/N_{0}\right)}_{\mathrm{eff},\mathrm{CTSSC}}^{\mathrm{inter}}}.$$

**Figure 3:**
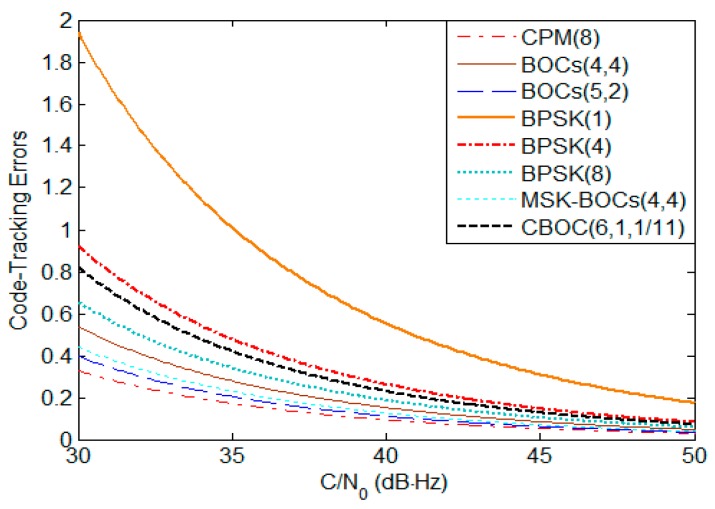
The code tracking errors of S band navigation signals.

To fairly determine the maximum and minimal power at receiver antenna output for each S band navigation signal, we assume their minimal power required to guarantee the same raw thermal noise pseudorange error of 0.2 m. Figure 3 depicts the code-tracking errors of S band navigation signals using noncoherent discriminator in white noise, where an early-late spacing of 0.1 chip, a receiver bandwidth of 16.363 MHz, and integration time of 4 ms are used to produce the results. The corresponding minimal power can be read back from Figure 3 for a particular code noise of 0.2 m, where the noise density $N_{0}$ is considered to be −204 dBW/Hz. For the assessment of worst-case interference effects, a maximum threshold of −126 dBW/m^2^ in S band and 0 dBi receiving antenna are used for Globalstar and the effective area of receiving antenna is estimated approximately as 29.4 dBm^2^ by

(29) $$A=\frac{G\lambda^{2}}{4\pi },$$

where the G is antenna gain and λ is carrier wavelength. Table 4 summarizes the minimal and maximum power of all above S band signals, where a margin of 3 dB has been considered between the maximum and minimal received powers. 

To evaluate radio frequency compatibility of S band navigation signals for future BDS, the worst results of compatibility between BDS candidate signals and other systems in S band based on the $\Delta {\left(C/N_{0}\right)}_{\mathrm{eff}}$ for signal acquisition and code tracking are presented as follows:

Case 1: Galileo candidate CBOC(6,1,1/11) is interfered by BDS candidate signals, i.e.,

(30) $$\{\begin{array}{l} I_{\mathrm{intra}}=I_{\mathrm{CBOC}\left(6,1,1/11\right),\mathrm{others}}^{\mathrm{Galileo}}\\ I_{\mathrm{inter}}=I_{\mathrm{candidate}}^{\mathrm{BDS}} \end{array},$$

where $I_{\mathrm{candidate}}^{\mathrm{BDS}}$ denotes the equivalent PSD of BDS candidate interfering signals, and the BDS candidate is assumed respectively as CPM(8), BOCs(4,4), BPSK(8) and MSK-BOCs(4,4).

**Figure 4:**
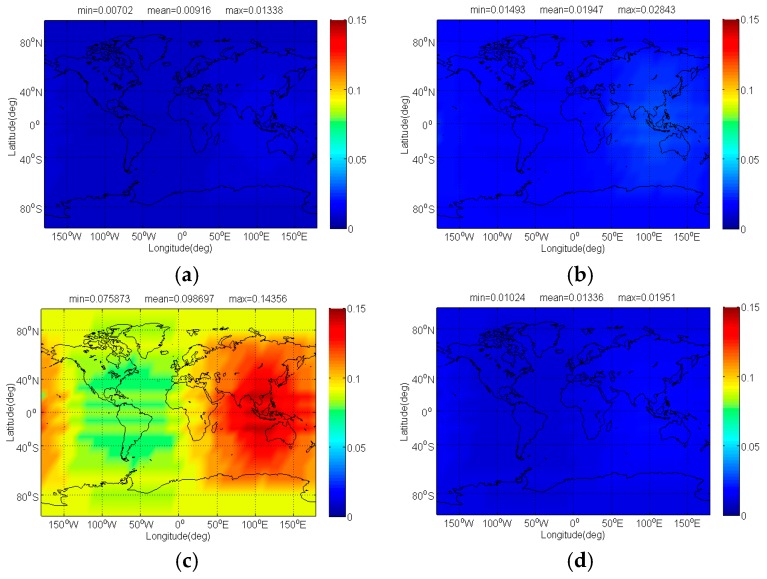
The $\Delta {\left(C/N_{0}\right)}_{\mathrm{eff},\mathrm{SSC}}^{\mathrm{inter},\mathrm{worst}}$ of Galileo candidate CBOC(6,1,1/11) in dB level interfered by BDS: (**a**) CPM(8); (**b**) BOCs(4,4); (**c**) BPSK(8); (**d**) MSK-BOCs(4,4).

**Figure 5:**
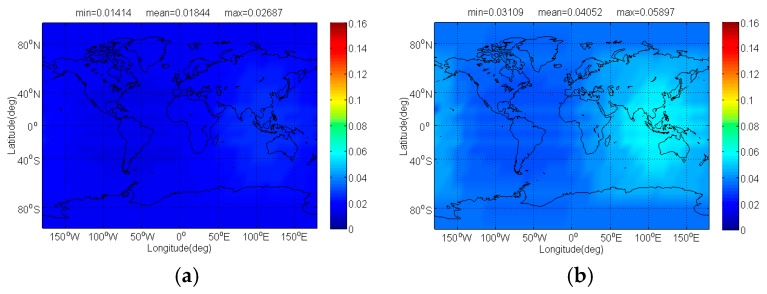

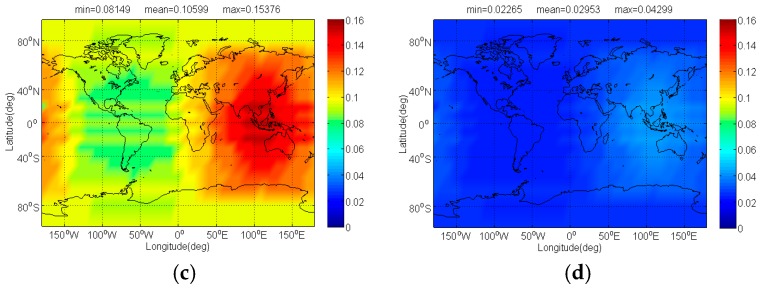
The $\Delta {\left(C/N_{0}\right)}_{\mathrm{eff},\mathrm{CTSSC}}^{\mathrm{inter},\mathrm{worst}}$ of Galileo candidate CBOC(6,1,1/11) in dB level interfered by BDS: (**a**) CPM(8); (**b**) BOCs(4,4); (**c**) BPSK(8); (**d**) MSK-BOCs(4,4).

The $\Delta {\left(C/N_{0}\right)}_{\mathrm{eff},\mathrm{SSC}}^{\mathrm{inter},\mathrm{worst}}$ and $\Delta {\left(C/N_{0}\right)}_{\mathrm{eff},\mathrm{CTSSC}}^{\mathrm{inter},\mathrm{worst}}$ of Galileo candidate CBOC(6,1,1/11) interfered by BDS candidate signals for signal acquisition and code tracking are respectively shown in Figure 4 and Figure 5. As shown, the mean $\Delta {\left(C/N_{0}\right)}_{\mathrm{eff},\mathrm{SSC}}^{\mathrm{inter},\mathrm{worst}}$ between CBOC(6,1,1/11) and CPM(8), BOCs(4,4), BPSK(8) and MSK-BOCs(4,4) at global scale are around 0.0092 dB, 0.0195 dB, 0.0987 dB and 0.0134 dB respectively, which means that compared to other candidates the CPM(8) introduces minimal interference on CBOC(6,1,1/11) in acquisition. Also, the CPM(8) is more superior in code tracking, with a smallest mean degradation of 0.0184 dB on CBOC(6,1,1/11). This result comes as no surprise because CPM(8) has comparable or better SSC and CTSSC with CBOC(6,1,1/11) as well as less maximum received power than other BDS candidates at worst situation. 

Case 2: Galileo candidate BPSK(1) is interfered by BDS candidate signals, i.e.,

(31) $$\{\begin{array}{l} I_{\mathrm{intra}}=I_{\mathrm{BPSK}\left(1\right),\mathrm{others}}^{\mathrm{Galileo}}\\ I_{\mathrm{inter}}=I_{\mathrm{candidate}}^{\mathrm{BDS}} \end{array}.$$

**Figure 6:**
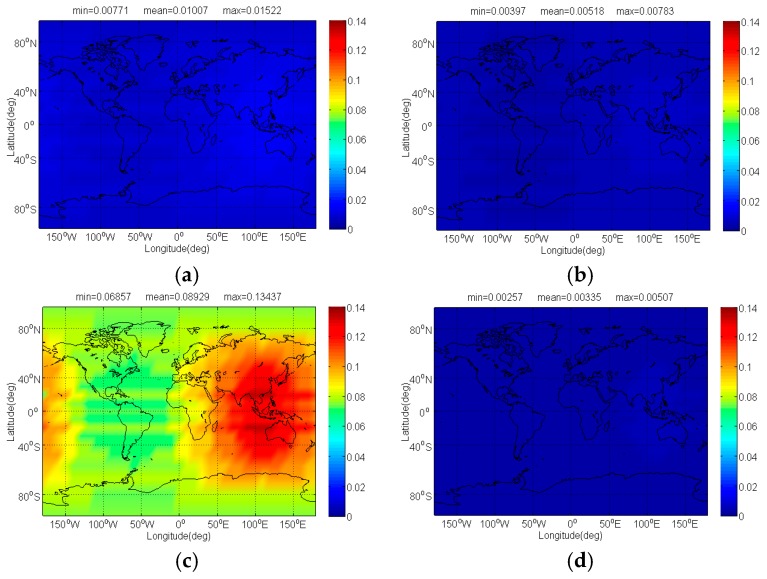
The $\Delta {\left(C/N_{0}\right)}_{\mathrm{eff},\mathrm{SSC}}^{\mathrm{inter},\mathrm{worst}}$ of Galileo candidate BPSK(1) in dB level interfered by BDS: (**a**) CPM(8); (**b**) BOCs(4,4); (**c**) BPSK(8); (**d**) MSK-BOCs(4,4).

**Figure 7:**
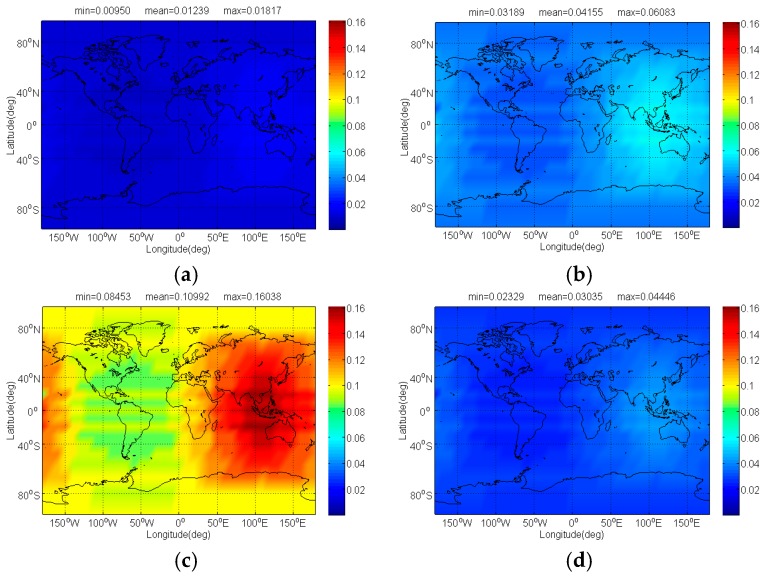
The $\Delta {\left(C/N_{0}\right)}_{\mathrm{eff},\mathrm{CTSSC}}^{\mathrm{inter},\mathrm{worst}}$ of Galileo candidate BPSK(1) in dB level interfered by BDS: (**a**) CPM(8); (**b**) BOCs(4,4); (**c**) BPSK(8); (**d**) MSK-BOCs(4,4).

Figure 6 and Figure 7 respectively show the $\Delta {\left(C/N_{0}\right)}_{\mathrm{eff},\mathrm{SSC}}^{\mathrm{inter},\mathrm{worst}}$ and $\Delta {\left(C/N_{0}\right)}_{\mathrm{eff},\mathrm{CTSSC}}^{\mathrm{inter},\mathrm{worst}}$ of Galileo candidate BPSK(1) interfered by BDS candidate signals. In previous analysis, we know that MSK-BOCs(4,4) has better SSC of −80.32 dB with BPSK(1), which implies that they allow peaceful coexistence with minimal mutual interference in signal acquisition. A perfect agreement can be observed in Figure 6, indicating that the mean $\Delta {\left(C/N_{0}\right)}_{\mathrm{eff},\mathrm{SSC}}^{\mathrm{inter},\mathrm{worst}}$ of BPSK(1) induced by MSK-BOCs(4,4) is around 0.006 dB, 0.001 dB and 0.08 dB smaller than those induced by CPM(8), BOCs(4,4) and BPSK(8) respectively. In code tracking, it is shown from Figure 7 that CPM(8) results in the minimal mean $\Delta {\left(C/N_{0}\right)}_{\mathrm{eff},\mathrm{CTSSC}}^{\mathrm{inter},\mathrm{worst}}$ of 0.01239 dB on Galileo BPSK(1) that are approximately 0.029 dB, 0.098 dB and 0.018 dB smaller than BOCs(4,4), BPSK(8) and MSK-BOCs(4,4), because CPM(8) has the smallest CTSSC at worst situation clearly shown in Figure 2d.

Case 3: Galileo candidate BPSK(4) is interfered by BDS candidate signals, i.e.

(32) $$\{\begin{array}{l} I_{\mathrm{intra}}=I_{\mathrm{BPSK}\left(4\right),\mathrm{others}}^{\mathrm{Galileo}}\\ I_{\mathrm{inter}}=I_{\mathrm{candidate}}^{\mathrm{BDS}} \end{array}.$$

Case 4: Galileo BPSK(8) is interfered by BDS candidate signals, i.e.,

(33) $$\{\begin{array}{l} I_{\mathrm{intra}}=I_{\mathrm{BPSK}\left(8\right),\mathrm{others}}^{\mathrm{Galileo}}\\ I_{\mathrm{inter}}=I_{\mathrm{candidate}}^{\mathrm{BDS}} \end{array}.$$

**Figure 8:**
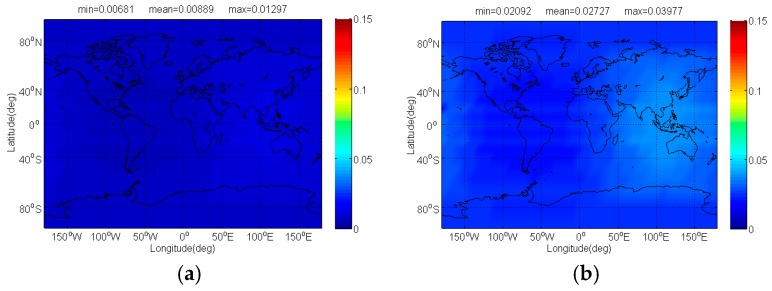

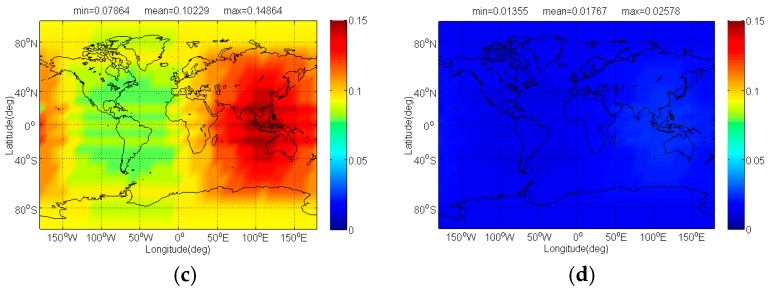
The $\Delta {\left(C/N_{0}\right)}_{\mathrm{eff},\mathrm{SSC}}^{\mathrm{inter},\mathrm{worst}}$ of Galileo candidate BPSK(4) in dB level interfered by BDS: (**a**) CPM(8); (**b**) BOCs(4,4); (**c**) BPSK(8); (**d**) MSK-BOCs(4,4).

**Figure 9:**
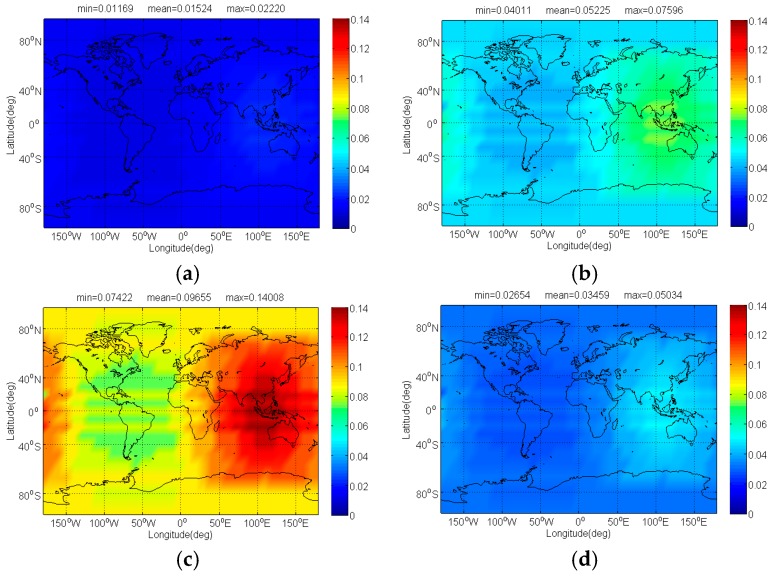
The $\Delta {\left(C/N_{0}\right)}_{\mathrm{eff},\mathrm{CTSSC}}^{\mathrm{inter},\mathrm{worst}}$ of Galileo candidate BPSK(4) in dB level interfered by BDS: (**a**) CPM(8); (**b**) BOCs(4,4); (**c**) BPSK(8); (**d**) MSK-BOCs(4,4).

**Figure 10:**
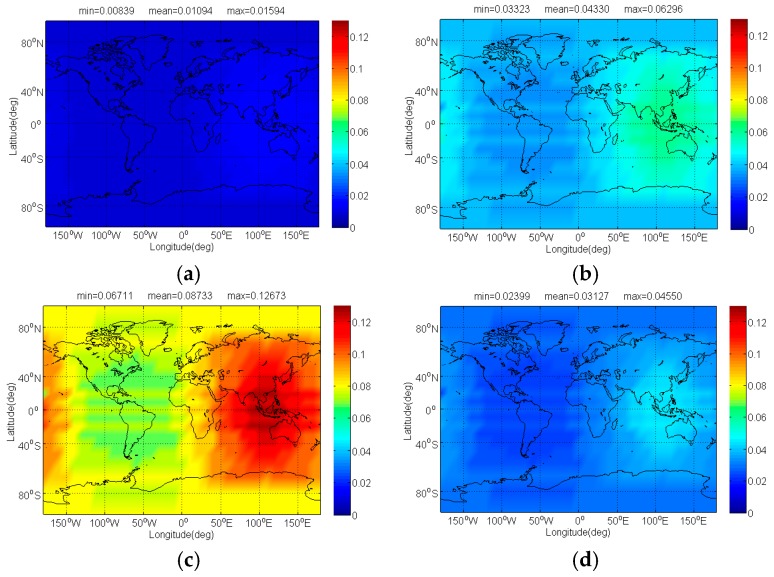
The $\Delta {\left(C/N_{0}\right)}_{\mathrm{eff},\mathrm{SSC}}^{\mathrm{inter},\mathrm{worst}}$ of Galileo candidate BPSK(8) in dB level interfered by BDS: (**a**) CPM(8); (**b**) BOCs(4,4); (**c**) BPSK(8); (**d**) MSK-BOCs(4,4).

**Figure 11:**
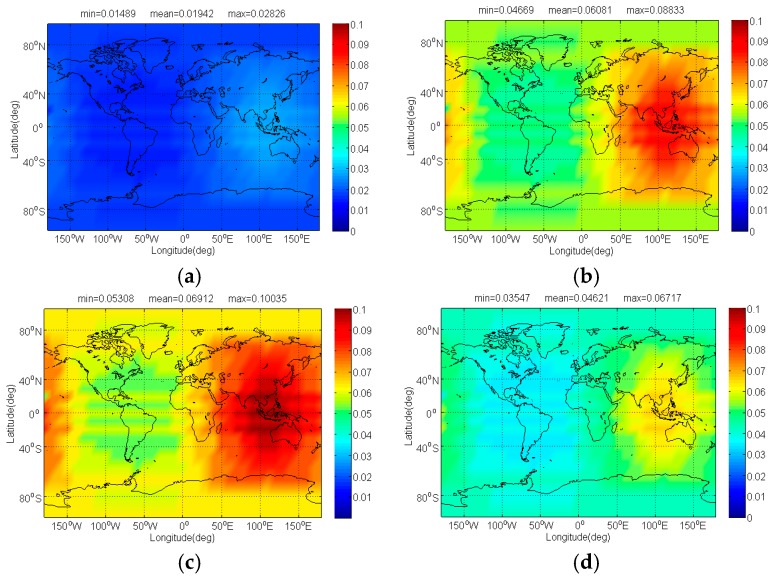
The $\Delta {\left(C/N_{0}\right)}_{\mathrm{eff},\mathrm{CTSSC}}^{\mathrm{inter},\mathrm{worst}}$ of Galileo candidate BPSK(8) in dB level interfered by BDS: (**a**) CPM(8); (**b**) BOCs(4,4); (**c**) BPSK(8); (**d**) MSK-BOCs(4,4).

The compatibility evaluation of Galileo candidate BPSK(4) and BPSK(8) interfered by BDS candidate signals in terms of $\Delta {\left(C/N_{0}\right)}_{\mathrm{eff},\mathrm{SSC}}^{\mathrm{inter},\mathrm{worst}}$ and $\Delta {\left(C/N_{0}\right)}_{\mathrm{eff},\mathrm{CTSSC}}^{\mathrm{inter},\mathrm{worst}}$ are respectively depicted in Figure 8, Figure 9, Figure 10 and Figure 11. Because of the better SSC and CTSSC of CPM(8) with BPSK(4) and BPSK(8) at worst situation, a conclusion can easily be drawn that the CPM(8) causes the less interference on Galileo candidate BPSK(4) and BPSK(8) in signal acquisition and code tracking than other BDS candidates. These figures also indicate that in worst case the mean performance degradations of BPSK(4) from CPM(8) are respectively at least 0.02 dB and 0.026 dB smaller than those from other BDS candidates for signal acquisition and code tracking, while CPM(8) respectively has more than 0.008 dB and 0.019 dB advantages over other BDS candidates in terms of mean $\Delta {\left(C/N_{0}\right)}_{\mathrm{eff},\mathrm{SSC}}^{\mathrm{inter},\mathrm{worst}}$ and $\Delta {\left(C/N_{0}\right)}_{\mathrm{eff},\mathrm{CTSSC}}^{\mathrm{inter},\mathrm{worst}}$ of BPSK(8) for global scale.

Case 5: IRNSS BOCs(5,2) is interfered by BDS candidate signals, i.e.

(34) $$\{\begin{array}{l} I_{\mathrm{intra}}=I_{\mathrm{BOCs}\left(5,2\right),\mathrm{others}}^{\mathrm{IRNSS}}+I_{\mathrm{BPSK}\left(1\right)}^{\mathrm{IRNSS}}\\ I_{\mathrm{inter}}=I_{\mathrm{candidate}}^{\mathrm{BDS}} \end{array}.$$

**Figure 12:**
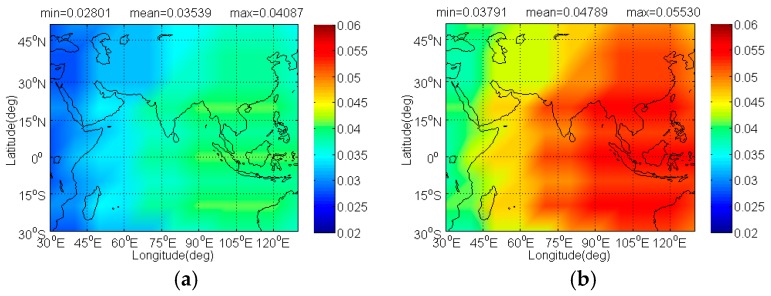

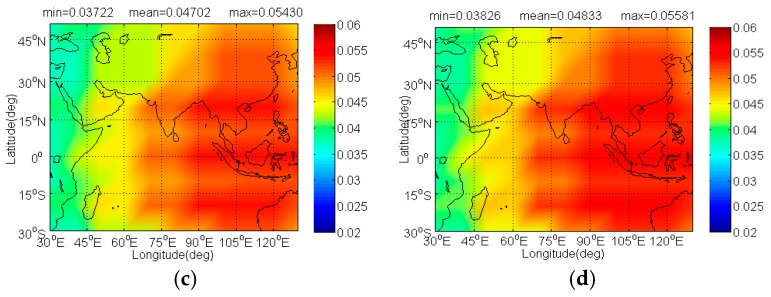
The $\Delta {\left(C/N_{0}\right)}_{\mathrm{eff},\mathrm{SSC}}^{\mathrm{inter},\mathrm{worst}}$ of IRNSS BOCs(5,2) in dB level interfered by BDS: (**a**) CPM(8); (**b**) BOCs(4,4); (**c**) BPSK(8); (**d**) MSK-BOCs(4,4).

**Figure 13:**
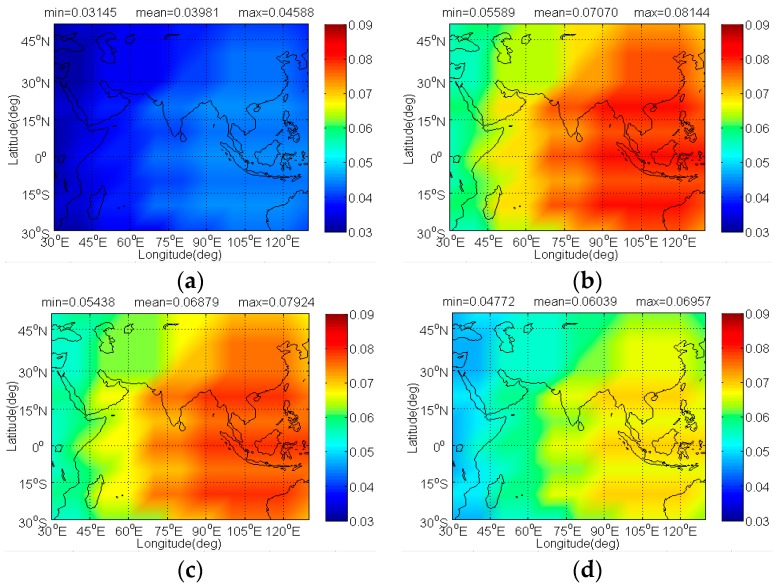
The $\Delta {\left(C/N_{0}\right)}_{\mathrm{eff},\mathrm{CTSSC}}^{\mathrm{inter},\mathrm{worst}}$ of IRNSS BOCs(5,2) in dB level interfered by BDS: (**a**) CPM(8); (**b**) BOCs(4,4); (**c**) BPSK(8); (**d**) MSK-BOCs(4,4).

The Figure 12 and Figure 13 illustrate the $\Delta {\left(C/N_{0}\right)}_{\mathrm{eff},\mathrm{SSC}}^{\mathrm{inter},\mathrm{worst}}$ and $\Delta {\left(C/N_{0}\right)}_{\mathrm{eff},\mathrm{CTSSC}}^{\mathrm{inter},\mathrm{worst}}$ of IRNSS BOCs(5,2) caused by BDS candidates. Since IRNSS is designed to provide positioning for India as well as the region extending up to 1500 km from its boundary, the compatibility analysis mainly focuses on the extended service area enclosed by the rectangle with latitude 30° S to 50° N and longitude 30° E to 130° E. As shown, the CPM(8) has the minimal $\Delta {\left(C/N_{0}\right)}_{\mathrm{eff},\mathrm{SSC}}^{\mathrm{inter},\mathrm{worst}}$ on IRNSS BOCs(5,2) with its mean 0.03539 dB, followed by BPSK(8), BOCs(4,4)and MSK-BOCs(4,4), at the same time, in code tracking the CPM(8) also behaves the smallest $\Delta {\left(C/N_{0}\right)}_{\mathrm{eff},\mathrm{CTSSC}}^{\mathrm{inter},\mathrm{worst}}$ on IRNSS BOCs(5,2) with its mean 0.03981dB, followed by MSK-BOCs(4,4), BPSK(8), and BOCs(4,4). 

Case 6: Single FDMA Globalstar signal is interfered by BDS candidate signals, i.e.

(35) $$\{\begin{array}{l} I_{\mathrm{intra}}=I_{\mathrm{SRRC},\mathrm{others}}^{\mathrm{Globalstar}}\\ I_{\mathrm{inter}}=I_{\mathrm{candidate}}^{\mathrm{BDS}} \end{array}.$$

**Figure 14:**
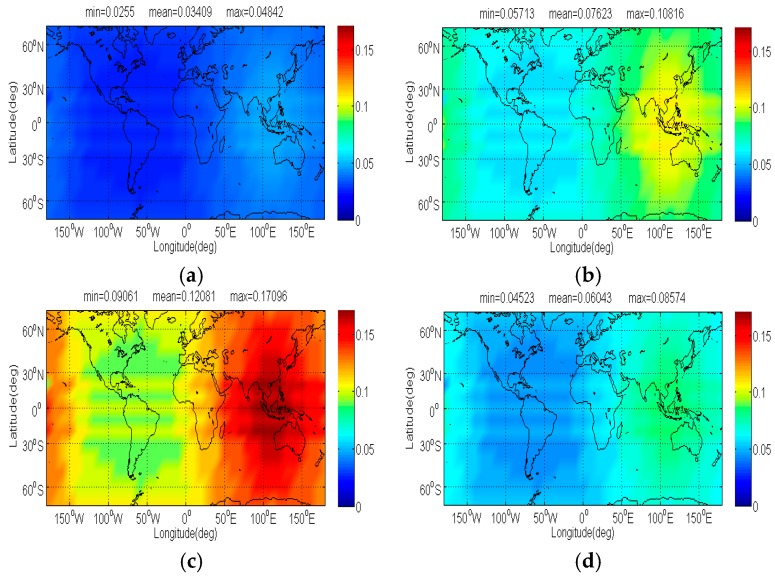
The $\Delta {\left(C/N_{0}\right)}_{\mathrm{eff},\mathrm{SSC}}^{\mathrm{inter},\mathrm{worst}}$ of single FDMA Globalstar signal in dB level interfered by BDS: (**a**) CPM(8); (**b**) BOCs(4,4); (**c**) BPSK(8); (**d**) MSK-BOCs(4,4).

**Figure 15:**
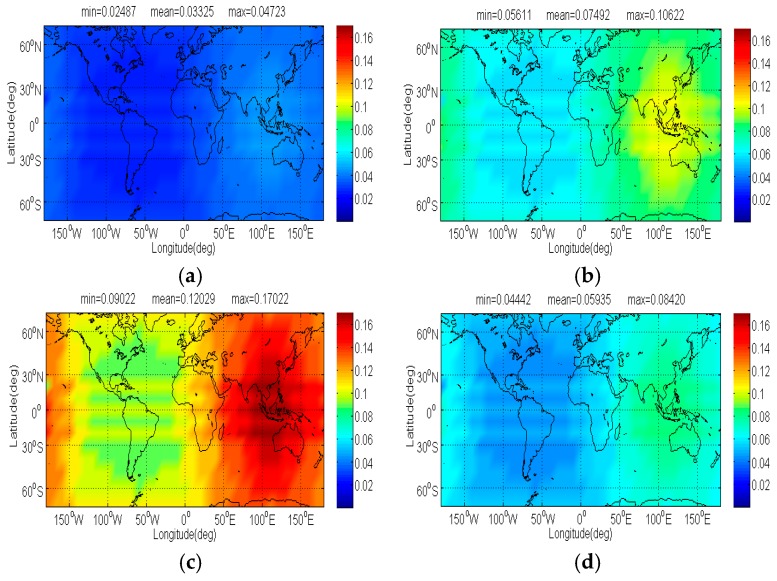
The $\Delta {\left(C/N_{0}\right)}_{\mathrm{eff},\mathrm{CTSSC}}^{\mathrm{inter},\mathrm{worst}}$ of single FDMA Globalstar signal in dB level interfered by BDS: (**a**) CPM(8); (**b**) BOCs(4,4); (**c**) BPSK(8); (**d**) MSK-BOCs(4,4).

The Figure 14 and Figure 15 show compatibility evaluation results between BDS and Globalstar. In real simulation, CPM(8) naturally entails less received power than other BDS candidates, together with comparable SSC and CTSSC, thus CPM(8) tends to introduce less interference on Globalstar for acquisition and code tracking shown clearly in Figure 14 and Figure 15. The figures indicate that CPM(8) has less $\Delta {\left(C/N_{0}\right)}_{\mathrm{eff},\mathrm{SSC}}^{\mathrm{inter},\mathrm{worst}}$ on *s*ingle FDMA Globalstar signal than BOCs(4,4), BPSK(8) and MSK-BOCs(4,4) at 0.0421 dB, 0.0867 dB and 0.0263 dB in average, whereas the mean $\Delta {\left(C/N_{0}\right)}_{\mathrm{eff},\mathrm{CTSSC}}^{\mathrm{inter},\mathrm{worst}}$ of *s*ingle FDMA Globalstar signal subjected by CPM(8) for code tracking is around 0.042 dB, 0.087 dB and 0.026 dB smaller than that induced by BOCs(4,4), BPSK(8) and MSK-BOCs(4,4).

Case 7: BDS candidate signals are interfered by IRNSS, Galileo candidate, and Globalstar, i.e.,

(36) $$\{\begin{array}{l} I_{\mathrm{intra}}=I_{\mathrm{candidate},\mathrm{others}}^{\mathrm{BDS}}\\ I_{\mathrm{inter}}=I_{\mathrm{SRRC}}^{\mathrm{Globalstar}}+I_{\mathrm{BOCs}\left(5,2\right)}^{\mathrm{IRNSS}}+I_{\mathrm{BPSK}\left(1\right)}^{\mathrm{IRNSS}}+I_{\mathrm{candidate}}^{\mathrm{Galileo}} \end{array}.$$

where $I_{\mathrm{candidate}}^{\mathrm{Galileo}}$ denotes the equivalent PSD of Galileo candidate interfering signals, and in this case the Galileo candidate is assumed as CBOC(6,1,1/11).

**Figure 16:**
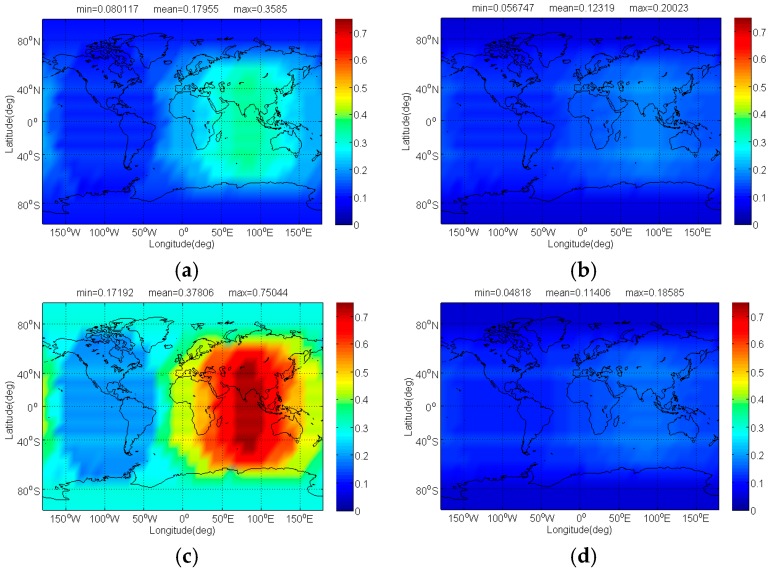
The $\Delta {\left(C/N_{0}\right)}_{\mathrm{eff},\mathrm{SSC}}^{\mathrm{inter},\mathrm{worst}}$ of BDS: (**a**) CPM(8); (**b**) BOCs(4,4); (**c**) BPSK(8); (**d**) MSK-BOCs(4,4) in dB level interfered by IRNSS, Galileo candidate, and Globalstar.

**Figure 17:**
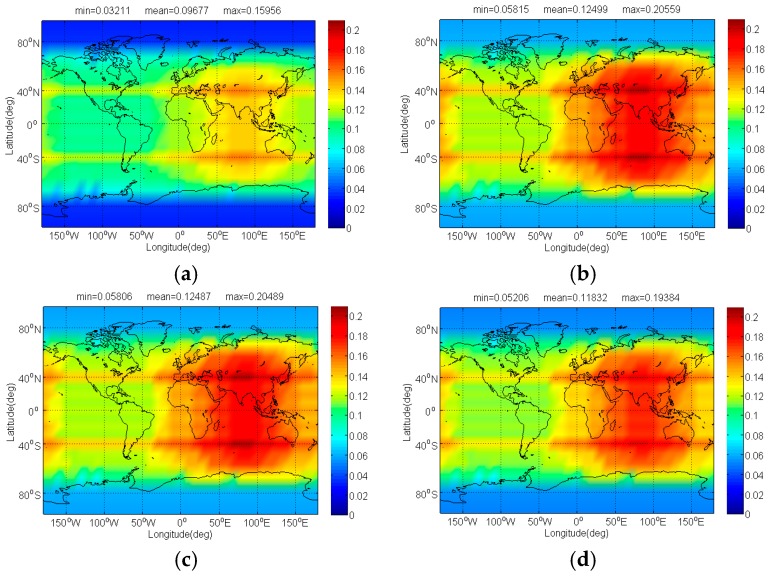
The $\Delta {\left(C/N_{0}\right)}_{\mathrm{eff},\mathrm{CTSSC}}^{\mathrm{inter},\mathrm{worst}}$ of BDS: (**a**) CPM(8); (**b**) BOCs(4,4); (**c**) BPSK(8); (**d**) MSK-BOCs(4,4) in dB level interfered by IRNSS, Galileo candidate, and Globalstar.

The compatibility evaluation of BDS candidates interfered by IRNSS, Galileo candidate, and Globalstar in terms of $\Delta {\left(C/N_{0}\right)}_{\mathrm{eff},\mathrm{SSC}}^{\mathrm{inter},\mathrm{worst}}$ and $\Delta {\left(C/N_{0}\right)}_{\mathrm{eff},\mathrm{CTSSC}}^{\mathrm{inter},\mathrm{worst}}$ are respectively depicted in Figure 16 and Figure 17. In signal acquisition, the MSK-BOCs(4,4) has minimal $\Delta {\left(C/N_{0}\right)}_{\mathrm{eff},\mathrm{SSC}}^{\mathrm{inter},\mathrm{worst}}$ induced by IRNSS, Galileo candidate and Globalstar, and its mean is 0.065 dB, 0.009 dB and 0.264 dB smaller than that of CPM(8), BOCs(4,4) and BPSK(8), which is mainly attributed to the best SSC of MSK-BOCs(4,4) with BPSK(1). In code tracking, IRNSS, Galileo candidate and Globalstar introduce less interference impacts on CPM(8), and the mean $\Delta {\left(C/N_{0}\right)}_{\mathrm{eff},\mathrm{CTSSC}}^{\mathrm{inter},\mathrm{worst}}$ of CPM(8) is over 0.02 dB smaller than that of MSK-BOCs(4,4), followed by BOCs(4,4) and BPSK(8).

From the compatibility evaluation results above, it is concluded that although the introduction of BDS S band candidate inevitably increases intersystem interference on existing or planned signals in same frequency band, the performance degradation is very little below 0.15 dB at the maximum. The effect of each BDS candidate caused by other systems in S band is less than 0.8 dB at the maximum. It even can be ignored. On the whole, the CPM(8) results in less mutual interference with most S band signals in acquisition and code tracking, followed by MSK-BOC(4,4), BOCs(4,4) and BPSK(8). Thus, CPM(8) is more superior as a future BDS signal solution in S band.

## 5. Conclusions

As the number of navigation and communication systems in operation or under development for S band increases, the signal mutual interference is getting very severe. Compatibility is a particularly essential issue to be considered for new S band signal design. The main purpose of this paper is to conduct a radio frequency compatibility evaluation and design a desired S band signal solution for future BDS, allowing peaceful coexistence of other systems with minimal mutual interference. Complete compatibility evaluation methods based on effective carrier-to-noise ratio degradation are described and derived from the perspective of output SNIR and code tracking error variance for both scenarios of acquisition and code tracking respectively. A real simulation, considering space constellations, modulation schemes and received power, is established to evaluate the compatibility of BDS candidates with Galileo candidate, IRNSS and Globalstar in S band. The worst-case results indicate that the introduction of BDS S band candidate causes very small degradation below 0.15 dB at the maximum on existing or planned signals in same frequency band, whereas the effect of each BDS candidate caused by other systems in S band is less than 0.8 dB at the maximum. It even can be ignored. Among these BDS candidates in S band, CPM(8) is better for minimizing the mutual interference, with most signals sharing the same frequency band for both scenarios of acquisition and code tracking. Thus, CPM(8) is a very desired S band signal solution for future BDS. This research provides a constructive reference for S band signal design for future BDS.

## Figures and Tables

**Table 1:** Space constellation parameters of BDS, IRNSS, Galileo and Globalstar.

Parameter	BDS	IRNSS	Galileo	Globalstar
Satellite Types	27MEO + 5GSO + 3IGSO	3GSO + 4IGSO	27MEO	32LEO
Constellation	5GSO: 58.75° E, 80° E, 110.5° E, 140° E, and 160° E; 3GSO: 118° E; 27MEO:Walker 27/3/1.	3GSO: 32.5° E, 83° E, and 131.5° E;2IGSO:55° E; 2IGSO:111.75° E;	Walker 27/3/1	32/8
Eccentricity	0°	0°	0°	0
Inclination	55°	29°	56°	52°
Semimajor Axis	GSO:42157.4 km; IGSO:42157.4 km; MEO:27899.4 km	GSO:42166.3 km; IGSO: 42166.3 km	MEO: 29601.3 km	LEO: 7785.4 km

**Table 2:** Simulation parameters and corresponding settings.

Simulation Parameter	Parameter Setting
Simulation Time	10 days
Time Resolution	1 min
Grid Resolution	Longitude: 10°; Latitude: 10°
Minimum Elevation Angle	10°
Receiver Bandwidth	Navigation signal: 16.363 MHz; Globalstar signal: 1.23 MHz

**Table 3:** The SSC of investigated signals with other interference signals in S band.

SSC(dB)		Interference Signals								
		CPM(8)	BOCs(4,4)	BPSK(8)	MSK-BOCs(4,4)	BPSK(1)	BPSK(4)	BOCs(5,2)	CBOC(6,1,1/11)	Globalstar
Desired Signals	CPM(8)	−70.54	−73.81	−74.36	−72.28	−73.24	−74.99	−70.62	−74.84	−72.16
	BOCs(4,4)	−73.81	−70.93	−72.17	−70.62	−79.93	−73.91	−73.10	−75.36	−72.72
	BPSK(8)	−74.36	−72.17	−70.90	−72.09	−69.32	−69.93	−74.98	−70.07	−72.48
	MSK-BOCs(4,4)	−72.28	−70.62	−72.09	−70.14	−80.32	−74.30	−71.60	−75.50	−72.08
	BPSK(1)	−73.24	−79.93	−69.32	−80.32	−61.86	−66.50	−77.89	−68.28	−72.04
	BPSK(4)	−74.99	−73.91	−69.93	−74.30	−66.5	−67.88	−78.37	−67.76	−72.26
	BOCs(5,2)	−70.62	−73.10	−74.98	−71.60	−77.89	−78.37	−69.36	−78.62	−72.86
	CBOC(6,1,1/11)	−74.84	−75.36	−70.07	−75.50	−68.28	−67.76	−78.62	−65.66	72.31
	${\mathrm{Globalstar}}_{\max}^{\mathrm{single}}$	−80.23	−80.51	−80.29	−80.03	−72.71	−77.36	−78.37	−77.52-	−83.13

**Table 4:** The minimal and maximum received power of all S band signals.

(dBW)	Maximum Received Power	Minimal Received Power
CPM(8)	−167.2	−170.2
BOCs(4,4)	−163.4	−166.4
BOCs(5,2)	−165.8	−168.8
CBOC(6,1,1/11)	−159.8	−162.8
BPSK(1)	−152.3	−155.3
BPSK(4)	−158.7	−161.7
BPSK(8)	−161.6	−164.6
MSK-BOCs(4,4)	−164.9	−167.9
Globalstar	−155.4	−158.4
